# Supplementary material for: Physiological and Transcriptomic Analysis Provides Insights into Low Nitrogen Stress in Foxtail Millet (Setaria italica L.)
Source: Int J Mol Sci. 2023 Nov 14;24(22):16321. doi: 10.3390/ijms242216321 (PMC10671652; doi:10.3390/ijms242216321)
Supplement: Supplementary file 1 [file ijms-24-16321-s001.zip › ijms-2694835-supplementary.pdf]

**Supplemental Table S1** The gene-specific primes for selected DEGs

| Gene ID | Primer | Sequence(5'—3') |
|---|---|---|
| 101753893 | Seita.3G110700-10G-qRTF | GGATGAAGCTCCTGCTCAAG |
| | Seita.3G110700-10G-qRTR | CGATGTTGCCGACGATGA |
| 101754330 | Seita.9G475300-11G-qRTF | GCCTGGAGCACATTAGCA |
| | Seita.9G475300-11G-qRTR | CCGTCGTAGAAGAACCTGAG |
| 101757408 | Seita.8G130200-28G-qRTF | GGTGGTGGACGATACCTTCA |
| | Seita.8G130200-28G-qRTR | CAGTGGTGTTGCTTCCCTTT |
| 101761991 | Seita.3G143100-22G-qRTF | CGCTCGCCTACTATGGTGA |
| | Seita.3G143100-22G-qRTR | CACACGCCCTGACTCAATC |
| 101764145 | Seita.3G014700-4G-qRTF | GCCGTTCCAGAAGTTCATCA |
| | Seita.3G014700-4G-qRTR | TCCTTGTTCCAGCTCCAGTA |
| 101767747 | Seita.5G396500-35G-qRTF | CCTCCTCATCTCCGTCATTG |
| | Seita.5G396500-35G-qRTR | GAGCAGCCAGTAGAAGTAGTC |
| 101771644 | Seita.5G238500-32G-qRTF | CGGTGTACGACAAGGAGAC |
| | Seita.5G238500-32G-qRTR | CACCTGTATGCGGGGAAG |
| 101772106 | Seita.3G034900-8G-qRTF | GGACTCCACCTTAGAAGATGACT |
| | Seita.3G034900-8G-qRTR | CTGCTGTTCCCACCGAATATC |
| 101772473 | Seita.6G159400-9G-qRTF | ACACGCTGGACCAGATCA |
| | Seita.6G159400-9G-qRTR | CGTTGACGCAGAAGTAGAGC |
| 101773925 | Seita.9G440900-2G-qRTF | CAGGCAGAAGGAGCAGTTC |
| | Seita.9G440900-2G-qRTR | TTGTTGACAGGCGTGTTGT |
| 101774390 | Seita.7G093500-14G-qRTF | CACCATCGGGAAGCAGAG |
| | Seita.7G093500-14G-qRTR | GTAGGCTCCGCACTTGTAG |
| 101775495 | Seita.8G170100-13G-qRTF | AAGGTGACGAACCGTGCTA |
| | Seita.8G170100-13G-qRTR | CCGTTGGTGTCGATCTTCTTG |
| 101776721 | Seita.8G200200-12G-qRTF | TGGCTGGAGCATGGAGTA |
| | Seita.8G200200-12G-qRTR | TGCTTGAACAGGACGAGTG |
| 101779143 | Seita.2G289500-20G-qRTF | CGGACGGCAAGTCATTCAT |
| | Seita.2G289500-20G-qRTR | GAGTTGAGCATCCAGGTGTAG |
| 101784607 | Seita.1G031400-33G-qRTF | GTGTTCGAGACCAAGGTAGC |
| | Seita.1G031400-33G-qRTR | TGATGTCCTCGTCCCTAACC |

**Supplemental Table S2** Differentially expressed genes (DEGs) of two foxtail millet genotypes under control and low nitrogen conditions.

| Part | Comparision | DEGs | Upregulated | Downregulated |
|---|---|---|---|---|
| Shoot | JG20LN vs JG20CK | 4256 | 2214 | 2042 |
| | JG22LN vs JG22CK | 1291 | 431 | 860 |
| | JG22CK vs JG20CK | 3581 | 1346 | 2235 |
| | JG22LN vs JG20LN | 1132 | 457 | 675 |
| Root | JG20LN vs JG20CK | 3191 | 1862 | 1329 |
| | JG22LN vs JG22CK | 1881 | 1115 | 766 |
| | JG22CK vs JG20CK | 3852 | 1547 | 2305 |
| | JG22LN vs JG20LN | 2028 | 932 | 1096 |

**Supplemental Table S3** Number and percentage of differentially expressed TFs between two different foxtail millet varieties in response to LN condition.

| TF terms | Shoot JG20LN vs JG20CK | Shoot Common | Shoot JG22LN vs JG22CK | Root JG20LN vs JG20CK | Root common | Root JG22LN vs JG22CK |
|---|---|---|---|---|---|---|
| bHLH | 18(12.5%) | 3(16.7%) | 6(16.2%) | 8(7.1%) | 1(3.0%) | 6(5.7%) |
| bZIP | 5(3.5%) | 1(5.6%) | 3(8.1%) | 7(6.2%) | 2(6.1%) | 3(2.9%) |
| DIVARICATA | 2(1.4%) | 1(5.6%) | 1(2.7%) | 2(1.8%) | 1(3.0%) | 2(1.9%) |
| ERF | 25(17.4%) | 1(5.6%) | 3(8.1%) | 22(19.5%) | 7(21.2%) | 23(21.9%) |
| GATA | 4(2.8%) | 0(0.0%) | 0(0.0%) | 3(2.7%) | 1(3.0%) | 2(1.9%) |
| HHO | 2(1.4%) | 1(5.6%) | 1(2.7%) | 1(0.9%) | 1(3.0%) | 2(1.9%) |
| Hsf | 5(3.5%) | 1(5.6%) | 3(8.1%) | 2(1.8%) | 1(3.0%) | 3(2.9%) |
| IBH(S)/HBP(R) | 2(1.4%) | 1(5.6%) | 1(2.7%) | 1(0.9%) | 1(3.0%) | 1(1.0%) |
| MADS | 4(2.8%) | 2(11.1%) | 4(10.8%) | 4(3.5%) | 0(0.0%) | 2(1.9%) |
| MYB | 12(8.3%) | 2(11.1%) | 6(16.2%) | 6(5.3%) | 1(3.0%) | 7(6.7%) |
| MYC | 1(0.7%) | 0(0.0%) | 0(0.0%) | 0(0.0%) | 0(0.0%) | 1(1.0%) |
| NAC | 1(0.7%) | 1(5.6%) | 1(2.7%) | 0(0.0%) | 0(0.0%) | 1(1.0%) |
| NF-Y | 6(4.2%) | 0(0.0%) | 0(0.0%) | 4(3.5%) | 3(9.1%) | 4(3.8%) |
| PCF | 3(2.1%) | 0(0.0%) | 0(0.0%) | 1(0.9%) | 1(3.0%) | 2(1.9%) |
| RAX | 2(1.4%) | 0(0.0%) | 0(0.0%) | 0(0.0%) | 0(0.0%) | 2(1.9%) |
| TCP | 2(1.4%) | 0(0.0%) | 0(0.0%) | 1(0.9%) | 0(0.0%) | 0(0.0%) |
| TGAL | 3(2.1%) | 0(0.0%) | 0(0.0%) | 8(7.1%) | 3(9.1%) | 3(2.9%) |
| Trihelix | 1(0.7%) | 0(0.0%) | 2(5.4%) | 2(1.8%) | 0(0.0%) | 7(6.7%) |
| WRKY | 29(20.1%) | 0(0.0%) | 0(0.0%) | 11(9.7%) | 6(18.2%) | 25(23.8%) |
| others | 17(11.8%) | 4(22.2%) | 6(16.2%) | 30(26.5%) | 4(12.1%) | 9(8.6%) |
| Total | 144(100%) | 18(100%) | 37(100%) | 113(100.0%) | 33(100.0%) | 105(100.0%) |

**Supplemental Table S4** The DEGs related with Starch and sucrose metabolism in two varieties.

| Parts | Shoots | | | | Roots | | | |
|---|---|---|---|---|---|---|---|---|
| Treatments | LNJG20-CKJG20 | | LNJG22-CKJG22 | | LNJG20-CKJG20 | | LNJG22-CKJG22 | |
| UP/DOWN | UP | DOWN | UP | DOWN | UP | DOWN | UP | DOWN |
| Gene ID | 101761744 | 101765726 | 101756806 | 101783222 | 101754475 | 101765726 | 101773502 | 101765726 |
| Gene ID | 101756806 | 101765746 | 101778895 | 101786399 | 101781847 | 101754626 | 101768458 | 101777643 |
| Gene ID | 101766064 | 101769822 | 101753890 | 101765726 | 101773502 | 101783222 | 101765746 | 105914669 |
| Gene ID | 101759340 | 101773215 | | 101773215 | 101781445 | 101784113 | 101786613 | 101784144 |
| Gene ID | 101760963 | 101756027 | | 101765746 | 101784519 | 101774056 | 101779998 | 101769336 |
| Gene ID | 101763600 | 101776290 | | 101774746 | 101775037 | 101755255 | 101770526 | 101784175 |
| Gene ID | 101755745 | 101766477 | | 101776688 | 101776290 | 101765110 | | 101786459 |
| Gene ID | 101758779 | 101775884 | | 101784175 | 101775239 | 101784144 | | 101774056 |
| Gene ID | 101755613 | 101776688 | | | 101786844 | 101752753 | | |
| Gene ID | 101786613 | 101754626 | | | 101772298 | 101759310 | | |
| Gene ID | 101778895 | 101752753 | | | | 101784175 | | |
| Gene ID | 101781208 | 101785561 | | | | 101774804 | | |
| Gene ID | 101781159 | 101755626 | | | | 101777786 | | |
| Gene ID | 101782849 | 101768057 | | | | 101754303 | | |
| Gene ID | 101753874 | 101768040 | | | | 101775414 | | |
| Gene ID | 101781445 | 101769477 | | | | 101786399 | | |
| Gene ID | 101784519 | 101774746 | | | | 101774746 | | |
| Gene ID | 101753890 | 101768017 | | | | 101768458 | | |
| Gene ID | 101754475 | 101760405 | | | | 101777643 | | |
| Gene ID | 101764238 | 101758109 | | | | | | |
| Gene ID | 105914669 | 101775414 | | | | | | |
| Gene ID | | 101782643 | | | | | | |
| Gene ID | | 101780809 | | | | | | |
| Gene ID | | 101786924 | | | | | | |
| Gene ID | | 101765386 | | | | | | |

**Supplemental Table S5** The DEGs related with photosynthesis in two varieties.

| Gene ID | Gene Item | LNJG20-CKJG20 | LNJG22-CKJG22 |
|---|---|---|---|
| 19526781 | PsaA | DOWN | DOWN |
| 101755060 | PsaK | DOWN | DOWN |
| 101759541 | PsaN | DOWN | DOWN |
| 101771677 | PsaO | DOWN | DOWN |
| 101779542 | PsaG | DOWN | DOWN |
| 101785215 | PsaH | DOWN | DOWN |
| 19526757 | PsbC | DOWN | DOWN |
| 101753687 | PsbQ | UP | DOWN |
| 101785134 | PsbA | DOWN | DOWN |
| 19526775 | b | UP | DOWN |
| 101767618 | LHC | DOWN | DOWN |

**Supplemental Table S6** The DEGs related with Plant hormone signal transduction in two varieties.

| gene_id | Gene Item | | Shoot | | Root | |
|---|---|---|---|---|---|---|
| | | | LNJG20-CKJG20 | LNJG22-CKJG22 | LNJG20-CKJG20 | LNJG22-CKJG22 |
| 101770000 | Auxin | AUX/IAA | DOWN | DOWN | DOWN | UP |
| 101763152 | | AUX/IAA | UP | DOWN | UP | UP |
| 101756773 | | AUX/IAA | UP | UP | UP | DOWN |
| 101786423 | | AUX/IAA | UP | UP | DOWN | UP |
| 101776449 | | AUX/IAA | UP | UP | UP | UP |
| 101776224 | | AUX/IAA | DOWN | UP | UP | UP |
| 101771394 | | AUX/IAA | DOWN | DOWN | DOWN | UP |
| 101776797 | | AUX/IAA | UP | UP | UP | UP |
| 101773098 | | AUX/IAA | DOWN | UP | UP | DOWN |
| 101782847 | | ARF | UP | UP | UP | UP |
| 101786550 | | ARF | UP | UP | UP | UP |
| 101759755 | | ARF | UP | UP | UP | UP |
| 101770279 | | GH3 | DOWN | DOWN | UP | UP |
| 101786612 | | GH3 | DOWN | DOWN | UP | UP |
| 101777525 | | GH3 | DOWN | DOWN | DOWN | UP |
| 101768288 | | GH3 | UP | UP | UP | UP |
| 101768539 | | SAUR | DOWN | DOWN | UP | UP |
| 101755080 | | SAUR | DOWN | DOWN | UP | UP |
| 101757474 | | SAUR | DOWN | UP | UP | UP |
| 101773025 | | SAUR | DOWN | UP | UP | UP |
| 101786592 | | SAUR | DOWN | UP | DOWN | DOWN |
| 101759405 | | SAUR | UP | DOWN | UP | UP |
| 101774716 | | SAUR | DOWN | UP | UP | UP |
| 101776313 | | SAUR | DOWN | DOWN | DOWN | DOWN |
| 101776714 | | SAUR | DOWN | UP | DOWN | DOWN |
| 101784781 | | SAUR | UP | DOWN | UP | UP |
| 101768762 | Cytokinine | AHP | UP | DOWN | UP | UP |
| 101759627 | | AHP | UP | UP | UP | UP |
| 101773981 | | AHP | UP | UP | UP | UP |
| 101782248 | | AHP | UP | UP | DOWN | DOWN |
| 101785369 | | B-ARR | UP | UP | UP | UP |
| 101781296 | | A-ARR | UP | UP | DOWN | UP |
| 101767490 | | A-ARR | UP | DOWN | UP | UP |
| 101778881 | | A-ARR | UP | UP | UP | UP |
| 101775649 | Gibberellin | TF | DOWN | UP | UP | UP |
| 101758377 | Ethylene | EBF1/2 | DOWN | DOWN | DOWN | DOWN |
| 101778576 | | EIN3 | UP | DOWN | DOWN | DOWN |
| 101766570 | | EIN3 | DOWN | DOWN | DOWN | DOWN |
| 101785783 | Abscisic acid | PYR/PYL | UP | UP | DOWN | UP |
| 101776342 | | PYR/PYL | UP | UP | UP | UP |
| 101786686 | | PYR/PYL | UP | UP | DOWN | UP |
| 101769449 | | PP2C | DOWN | DOWN | UP | DOWN |
| 101753735 | | PP2C | DOWN | DOWN | UP | DOWN |

| | | | | | |
|---|---|---|---|---|---|
| 101783265 | PP2C | DOWN | DOWN | DOWN | UP |
| 101768564 | PP2C | DOWN | DOWN | UP | DOWN |
| 101780602 | PP2C | DOWN | DOWN | DOWN | DOWN |
| 101764618 | SnRK2 | DOWN | DOWN | DOWN | DOWN |
| 101763654 | SnRK2 | DOWN | DOWN | DOWN | UP |
| 101775125 | SnRK2 | DOWN | DOWN | DOWN | DOWN |
| 101765115 | SnRK2 | UP | DOWN | UP | UP |
| 101757593 | ABF | DOWN | DOWN | UP | DOWN |
| 101776070 | ABF | DOWN | DOWN | DOWN | DOWN |
| 101778442 | ABF | DOWN | DOWN | UP | DOWN |
| 101758999 | ABF | UP | UP | UP | UP |

**Supplemental Table S7** The DEGs related with glycolysis in two varieties.

| gene_id | Gene Item | Shoot | | Root | |
|---|---|---|---|---|---|
| | | LNJG20-CKJG20 | LNJG22-CKJG22 | LNJG20-CKJG20 | LNJG22-CKJG22 |
| 101754626 | HXK | DOWN | UP | DOWN | DOWN |
| 101775414 | | DOWN | DOWN | DOWN | DOWN |
| 101784144 | | DOWN | UP | DOWN | DOWN |
| 101772215 | AEP | DOWN | DOWN | DOWN | UP |
| 101771409 | | UP | DOWN | UP | UP |
| 101754303 | GPI | UP | UP | DOWN | DOWN |
| 101778700 | ATP-PFK | DOWN | DOWN | DOWN | DOWN |
| 101780397 | | DOWN | DOWN | DOWN | DOWN |
| 101780059 | | DOWN | DOWN | UP | UP |
| 101756322 | | UP | UP | DOWN | UP |
| 101756917 | | UP | DOWN | DOWN | DOWN |
| 101786189 | FBA | UP | UP | UP | DOWN |
| 101756517 | | DOWN | DOWN | DOWN | DOWN |
| 101754112 | | DOWN | DOWN | DOWN | DOWN |
| 101760718 | TIM | UP | UP | UP | UP |
| 101785151 | PGK | DOWN | DOWN | DOWN | DOWN |
| 101768835 | | DOWN | DOWN | DOWN | DOWN |
| 101759360 | | DOWN | DOWN | DOWN | DOWN |
| 101785907 | | UP | UP | UP | UP |
| 101775268 | | DOWN | DOWN | DOWN | DOWN |
| 101783820 | | DOWN | DOWN | UP | UP |
| 101776203 | PGMP | DOWN | DOWN | DOWN | UP |
| 101763575 | | UP | UP | UP | DOWN |
| 101771740 | | DOWN | DOWN | DOWN | DOWN |
| 101757916 | | DOWN | UP | DOWN | DOWN |
| 101781167 | ENO | DOWN | DOWN | DOWN | UP |
| 101769604 | | DOWN | DOWN | DOWN | DOWN |
| 101783767 | PCK | UP | UP | UP | DOWN |
| 101768352 | PK | DOWN | DOWN | DOWN | DOWN |
| 101759427 | | DOWN | DOWN | DOWN | DOWN |
| 101769537 | | UP | DOWN | UP | UP |
| 101780488 | | DOWN | DOWN | DOWN | DOWN |
| 101784708 | | UP | UP | UP | UP |
| 101763108 | PDH-E1$\alpha$ | DOWN | DOWN | DOWN | UP |
| 101775698 | PDC | DOWN | DOWN | DOWN | DOWN |
| 101763713 | PDH-E2 | DOWN | DOWN | UP | UP |
| 101769555 | | UP | DOWN | UP | UP |
| 101756978 | DLD | DOWN | DOWN | UP | UP |
| 101782764 | ALDH | DOWN | DOWN | UP | UP |
| 101761749 | | UP | UP | UP | UP |
| 101755454 | | UP | UP | UP | DOWN |
| 101776963 | | UP | UP | DOWN | UP |
| 101762488 | | DOWN | UP | DOWN | UP |

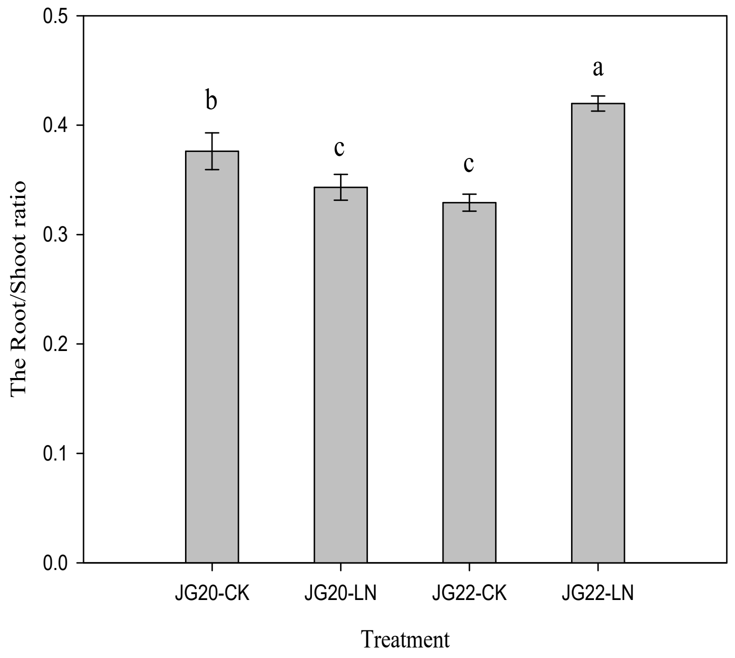


**Supplemental Figure S1** The root/shoot ratio in two foxtail millet varieties under control and low nitrogen conditions. JG20-CK, JG20 under control; JG20-LN, JG20 under low nitrogen; JG22-CK, JG22 under control; JG22-LN, JG22 under low nitrogen. Error bars represented standard error of three biological replicates; Lowercase indicates significance at the level of $P<0.05$.
